# Communication between Autonomous Vehicles and Pedestrians: An Experimental Study Using Virtual Reality

**DOI:** 10.3390/s23031049

**Published:** 2023-01-17

**Authors:** Symbat Zhanguzhinova, Emese Makó, Attila Borsos, Ágoston Pál Sándor, Csaba Koren

**Affiliations:** Department of Transport Infrastructure and Water Resources Engineering, University of Győr, Egyetem tér 1, 9026 Győr, Hungary

**Keywords:** autonomous vehicle, pedestrian, crossing, LED communication, virtual reality

## Abstract

One of the major challenges of autonomous vehicles (AV) is their interaction with pedestrians. Unofficial interactions such as gestures, eye contact, waving, and flashing lights are very common behavioral patterns for drivers to express their intent to give priority. In our research we composed a virtual reality experiment for a pedestrian crossing in an urban environment in order to test pedestrians’ reactions on an LED light display mounted on a virtual AV. Our main research interest was to investigate whether communication patterns influence the decision making of pedestrians when crossing the road. In a VR environment, four scenarios were created with a vehicle approaching a pedestrian crossing with different speeds and displaying a special red/green sign to pedestrians. Here, 51 persons participating in the experiment had to decide when crossing is safe. Results show that the majority of people indicated they would cross in the time windows when it was actually safe to cross. Male subjects made their decision to cross slightly faster but no significant differences were found in the decision making by gender. It was found that age is not an influencing factor, either. Overall, a quick learning process was experienced proving that explicit communication patterns are self-explaining.

## 1. Introduction

Globally, pedestrians and cyclists represent 26% of all road deaths [1]. Thanks to engineering measures and governmental initiatives, the number of traffic accidents is moderately decreasing in developed countries. Nevertheless, the share of accidents involving vulnerable road users remains high. The new era of autonomous vehicles (AV) will bring about new challenges as well as smart solutions, which pedestrians can presumably benefit from.

Vehicle automation is an extensively discussed topic nowadays. Perceiving the urban road environment is one of the major challenges in the autonomous vehicle industry. Numerous studies over the last ten years have concentrated on automatic driving for relatively straightforward road environments, such as highways [2]. One of the open questions is the way of communication between autonomous vehicles and pedestrians. Since pedestrians are the most vulnerable road users in traffic, visual eye-to-eye contact with drivers is now a necessary input for pedestrians to guarantee that the motorist has observed them crossing the road or detected their intention to cross [3]. Unofficial interactions such as gestures, eye contact, waving, and flashing lights are very common behavioral patterns for drivers to express their intent to give priority. Lately, various designs of external human–machine interfaces (eHMI) that increase the efficiency of interaction between AVs and road users are being tested throughout the world. Fully autonomous vehicles (FAVs) can indicate their intention to yield to pedestrians via eHMIs. By reducing the ambiguity concerning FAV intentions and increasing pedestrians’ initial trust and comprehension, they help in communication with pedestrians [4,5,6,7].

In our research we designed a virtual reality experiment for a pedestrian crossing in an urban environment. Its purpose was to test pedestrians’ reaction to an LED light display mounted outside a virtual autonomous car. Our main research interest was to investigate whether communication patterns influence the decision-making of pedestrians when crossing the road at a designated pedestrian crossing. More specifically, we were investigating how pedestrians react to red and green LED signals located on the front bumper of the vehicle. Although it is understood that AVs should stop immediately for pedestrians, it is not certain if pedestrians will react or whether they will understand and react to an approaching AV. Even in the later phase of AV market penetration, it might not be obvious for the pedestrians whether the approaching vehicle is (a) a human-driven one or (b) an AV in autonomous mode or (c) an AV in human driven mode.

The following section presents a literature review on pedestrian/AV interaction and the process of communication between them. Section 3 introduces the VR environment we developed, interaction scenarios, and the questionnaire survey. Section 4 contains results of the simulations, surveys, and observations. Finally, in Section 5 are the discussion and conclusions.

## 2. Literature Review

By 2030, it is predicted that autonomous vehicles will be a reliable and economical form of transportation, gradually replacing human-driven vehicles in the process. Besides improving the overall effectiveness, convenience, and safety of our roads and transportation network, these new capabilities could have significant global impacts that could radically change civilization as we know it [8,9].

One aspect of that process is the changing dynamic between vehicles and pedestrians. The communication between vulnerable users and vehicles should follow traffic safety rules, however informal interactions, such as eye contact, gestures, waving, etc., cannot be easily codified. These interactions are frequently used to promote communication between cars and vulnerable users. However, as autonomous cars will be introduced into the transportation system, drivers will become less involved in the decision process of driving. The pedestrian will then have to interact with a vehicle that is no longer driven by a human, which will require a new kind of informal communication [10,11,12].

To enhance safety and efficiency for all road users in this unique traffic environment, the autonomous vehicle must provide unambiguous signals to pedestrians to inform them of the vehicle’s status and intent [13].

Specifically, the most problematic scenario will be the perception of information provided by AVs. To improve the effectiveness and safety of such interactions, an external human–machine interface (eHMI) might be used to display dynamic information. However, an effective eHMI must evolve from the cues currently used by drivers and pedestrians to communicate and negotiate their interactions [14,15,16].

Madigan et al. [17] conducted a pedestrian simulator experiment with thirty-eight participants. The key factors included were: the presence or absence of a zebra crossing, different AV approach directions, yielding or non-yielding AV behavior, and presence or absence of a light-based eHMI. Where there was no zebra crossing, the eHMI had the greatest impact since it encouraged earlier crossing due to pedestrian confidence in the signal. The results of this study showed the importance of eHMI as an explicit communication in road user–AV interaction for situations where there is a doubt about an AV’s right-of-way in relation to other road users.

There are arguments about the various forms of eHMIs that can be used to enhance human decision making when crossing. For instance, de Clercq et al. [14] used various eHMI types such as front brake lights, Knightrider animation, smiley, and text [WALK] that fostered pedestrians to cross. According to the feel-safe percentages for yielding vehicles, the front brake lights, Knightrider, smiley, and text were higher than baseline. Contrary to the three other eHMIs, the text eHMI did not require any learning. There are several authors [18] following the same principle and confirming the benefits of sending large-scale text messages from cars to pedestrians. A disadvantage of text-based eHMIs is that they would need to be translated into many different languages, whereas anthropomorphic eHMIs pose the challenges of cost and durability [2].

A few other eHMI prototypes were developed for AVs by Guo et al. [19], such as text, arrowed (dynamic), text and symbol, symbol only, tick and cross, and traffic lights, along with two sub-designs (cross and do not cross). The results showed that for 65.1% of the participants, external communication would have a positive effect on their decision-making and 68.5% of participants felt safe while seeing eHMI ‘green’, ‘text’, ‘symbol’, or ‘dynamic’.

In addition to the various eHMI designs and positioning on the AVs, there are studies that explored the interaction in different traffic situations (signalized and unsignalized). In their study, Jayaraman et al. [20] manipulated two variables: AV driving behavior (defensive, normal, and aggressive) and traffic signalization (signalized and unsignalized). The results showed that depending on the type of crossing, aggressive driving reduces faith in AVs. In a VR study by Nunez Velasco et al. [21], pedestrians were asked to choose whether or not to cross the road after being shown a series of scenarios through movies played on a head-mounted display (HMD). According to the results, a zebra crossing and a greater separation between the pedestrian and the AV increased the pedestrian’s intention to cross. Results from Havard and Willis [22] concurred with these findings confirming that pedestrians’ perceptions of safety, convenience, and vulnerability are positively affected by zebra crossings. The findings of these studies demonstrate that the existence of supporting traffic infrastructure, such as a zebra crossing, can affect pedestrians’ levels of trust and readiness to cross in front of AVs.

Researchers have explored the impact of LED color on pedestrian decision making. Dey et al. [23] investigated the effectiveness of light band eHMIs with three colors (green, red, and cyan); the results indicated that green color was preferred, while red was equated with stopping intentions. On the other hand, cyan can stand as a neutral color and may therefore be a better choice for conveying a yielding message. According to SAE International’s recommended practice [24], blue–green marker lamps should be used to show the engagement of the (automated driving systems) ADS.

Faas and Baumann [25] compared the efficiency of flashing, steady, and sweeping light signals to convey the AV’s intention to yield. In contrast to sweeping signals, flashing signals encouraged pedestrians to cross more quickly, improving traffic flow.

Elderly pedestrians are particularly at higher risk due to their slower reaction time and reduced mobility. Mason et al. [26] conducted a survey of elderly pedestrians walking near cars backing out of parking lot spaces. According to the study, when elderly people are substantially more likely to see a second projected message on the ground behind the car than they are to notice the brake lights that are already there.

The majority of studies concluded that implicit communication remains dominant and advised against text-based and instructive eHMIs [2]. In the study of Madigan et al. [17], some participants expressed uncertainty about the eHMI’s meaning, which emphasizes that any light-based communication tool should be explained in advance. Roughly a quarter of the participants had no idea what the eHMI meant, demonstrating the impossibility of learning or understanding this kind of communication through intuition. Dey and Terken [27] stressed that explicit communication such as eye-contact and gestures between human-driven vehicles and pedestrians is not mandatory or critically important in common traffic situations.

In summary, it is still uncertain whether eHMIs can be used or intuitively grasped by pedestrians. Finding the ideal eHMI modality is a research area that has great potential. For our research, we formulated the following research question: *How do LED signals of red/green color and the speed of AVs influence the decision making of pedestrians at a designated pedestrian crossing?* In the current paper, the results of the first in a series of experiments are summarized.

## 3. Method

The procedure for our experiment was as follows:

1. Set up a 360° space using an existing urban designated pedestrian crossing.
2. Set up the VR environment and create the four scenarios.
3. Give instructions to the subjects participating in the experiment.
4. Show the scenarios to the subjects one by one.
5. Administer the questionnaire survey.

The process of setting-up the VR space, the scenarios, and the questionnaire survey are explained in the following section.

### 3.1. VR Space

The selected location is a pedestrian crossing at the intersection of a two-lane main road and a local street in Győr (47°40′14.6″ N 17°38′33.0″ E). To create the 360° space, we used a Kandao Obsidian Go 360° camera to take several high-resolution photos, which were later stitched together to reconstruct an exact replica of the location. Pictures were taken under good weather conditions in the early morning hours avoiding heavy traffic, which made it easier to take noise-free pictures. Six 180° photos were taken at several points and stitched together by the Kandao Studio software to generate an 8K resolution photo. This photo provided the texture needed to create the virtual space for the modeling process.

We decided to deform a basic mesh until it attained the desired shape. We cut a sphere in half using the Blender 3D open source modeling software, subdivided according to the resolution of the photo, and dragged our photo onto the sphere as a texture. We inverted this texture so that it does not look outward from the sphere, but towards its center, giving the viewer a natural image. The surface of the sphere was equipped with a shadow catcher feature, so that the objects inside can cast a shadow on its surface, resulting in a more natural space.

The model of a vehicle equipped with autonomous functions (Nissan Leaf) developed by the Vehicle Industry Research Center of the university was used in Blender [28]. As part of the modeling, the completed model and its objects were provided with photorealistic textures and movement animations.

The simulation used was principled BDSF (bidirectional scattering distribution function) and photo textures were applied to the models. As a result, we had a solid and constant 60 FPS (frames per second) playback speed even during the simulation in Blender.

From the beginning of 2022, within the list of Blender add-ons, both the VR and XR add-ons, which can be used for examining 3D spaces, are publicly available. We simply enabled the VR add-on by properly selecting the correct preferences and from then on, the application communicated with the steamVR application, within which the simulations started automatically when the animation was played. With the help of the accessory, we were able to adjust the height, rotation angle, position, and other properties of the camera in relation to the height of the test subjects so we were able to adapt to the physical characteristics of each test subject.

**Figure 1:**
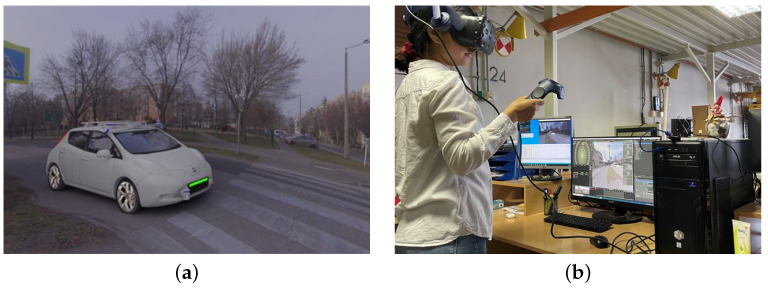
Simulation (**a**) Model of the AV in the 3D environment. (**b**) The experiment in process.

Our VR hardware, an HTC Vive Pro 2 proved to be an excellent tool for testing the behavior of experimental subjects, it was possible to integrate it into the software used. We had the opportunity to record what the test subjects saw inside the glasses during the simulations and monitor it in real time. If there was a problem due to hardware overload, we could make corrections and adjustments immediately (Figure 1). In order to track when the subjects would decide to cross, an Arduino Uno microcontroller was mounted on their leg to indicate when they initiated their crossing walk.

### 3.2. Scenarios

The subjects were instructed to indicate when they would be ready to cross the road at the designated pedestrian crossing by moving their leg. The micro-controller mounted on their right leg gave an indication when they made this decision.

Altogether four scenarios were created as follows:

1. Scenario 1: This scenario was 24 s long, where the AV approached the pedestrian crossing at a speed of 15 km/h, LED signal was red throughout. The AV did not stop and yield, it was safe to cross the pedestrian crossing after the vehicle left, this was after 14.3 s.
2. Scenario 2: This scenario was 24 s long, where the AV approached the pedestrian crossing at a speed of 15 km/h, with a red LED signal. At 6.3 s, the vehicle made a stop at the pedestrian crossing and the LED signal turned green. At 12 s, the LED turned red and the AV started to move again. It was safe to cross in between 6.3 and 12 s, and technically also after the back of the vehicle left the pedestrian crossing, which took place at 15.9 s.
3. Scenario 3: This scenario is similar to Scenario 1, with the exception that it was 12 s long, and that the speed of the vehicle was 30 km/h, LED signal was red throughout. It was safe to cross the pedestrian crossing after 4.1 s, when the vehicle left the area of the pedestrian crossing.
4. Scenario 4: This scenario is similar to Scenario 2, however it was 12 s long, and the AV’s speed was 30 km/h. After 3.2 s, the vehicle made a stop at the pedestrian crossing and the LED signal turned green. At 6.1 s, the LED turned red and the AV started to move again. It was safe to cross in between these two time instants, but also after the back of the vehicle left the crossing, which took place at 7.7 s.

**Figure 2:**
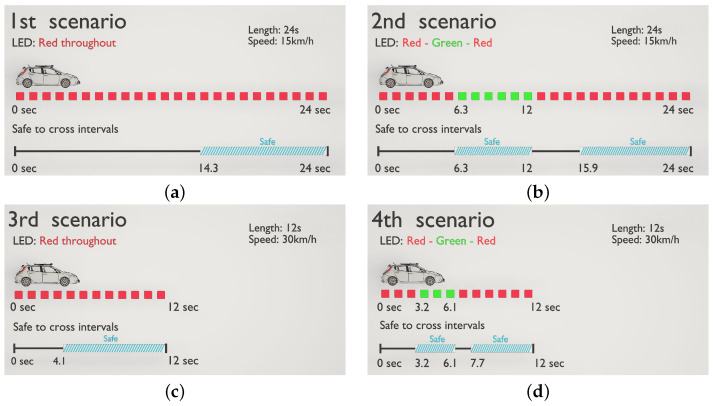
Scenarios: (**a**) Scenario 1, (**b**) Scenario 2, (**c**) Scenario 3, (**d**) Scenario 4.

A visual illustration of the scenarios is given in Figure 2.

### 3.3. Survey

Participants filled out a questionnaire survey once they completed the experiment. This questionnaire requested demographic information such as participant’s age, gender, nationality, education, one question about how they judge their behaviour (very cautious, moderately cautious, confident) at a pedestrian crossing, and one question about their trust in LEDs.

This latter question offered three possible answers:

1. I cannot imagine relying on this kind of equipment, only if the car has stopped am I willing to cross (limited trust in LED light display).
2. After some time of getting familiar with the LED, I think I would rely on it and cross the road when seeing the green light (moderate trust in LED light display).
3. The message was clear, I would cross the road without hesitating (trust in LED light display).

All the statistical analyses were conducted in R [29].

## 4. Results

The experiment was conducted in the Road VR lab of the University of Győr, Hungary. Participants received detailed instructions about the process of the experiment and before the actual scenarios they could also experience the VR environment. They were instructed that they would be standing at a pedestrian crossing, would need to observe a driverless car coming from their left, and once they felt it safe to cross they should indicate that by moving their right leg forward (sensed by the micro-controller). A few of the participants experienced motion sickness (especially the elderly). For them it was also possible to do the experiment while seated.

The four scenarios were shown one by one, after which the participants were asked to fill out the questionnaire. The entire experiment took about 20 min to complete. Fifty-one participants (24 female, 27 male) participated in the experiment in the 15–70 age range (mean = 39.11, standard deviation = 20.80). Furthermore, 82% of participants were Hungarian citizens, and the rest were internationals.

**Figure 3:**
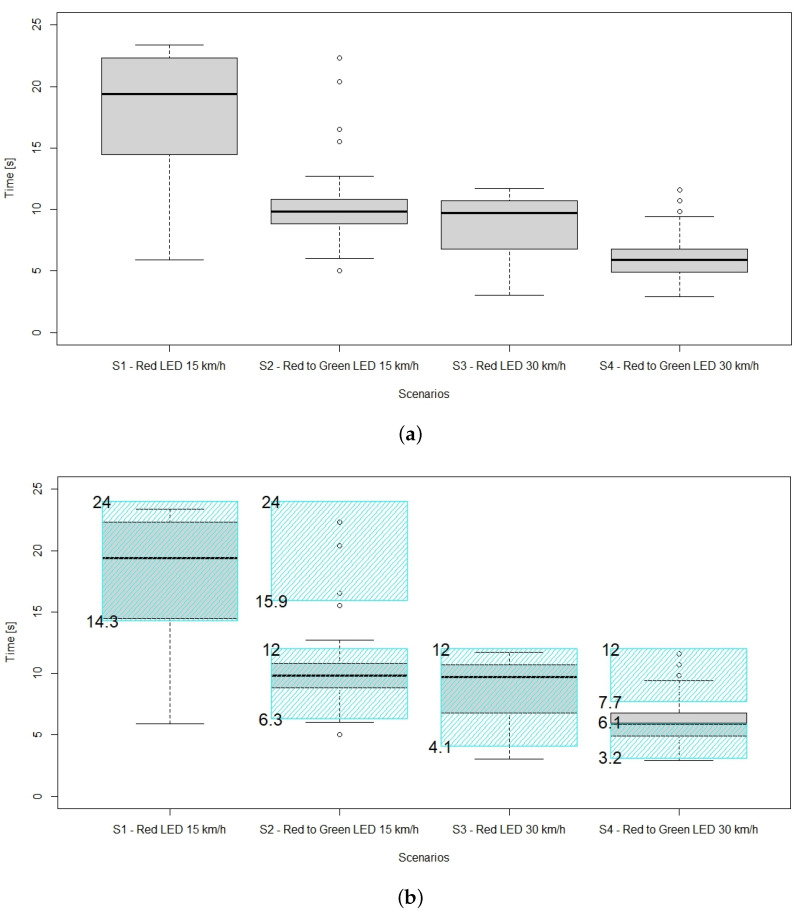
Boxplots of time required to make the decision to cross. (**a**) Distribution per scenario. (**b**) Safe to cross regions (shaded areas complying with scenarios in Figure 2).

During the experiment, the time at which subjects moved their right leg was observed; this point in time was measured from the very beginning of the scenario. In Figure 3, these time values are shown in a boxplot. In this plot, horizontal lines show the minimum, first quartile, median, third quartile, and maximum, whereas small circles plotted were considered outliers. The shaded areas show the time windows where it is safe to cross. As for scenarios 1 and 3, this was after the vehicle left, and as for scenarios 2 and 4, it was when the vehicle first stopped in front of the pedestrian crossing, then later as the vehicle left the pedestrian crossing. In Table 1, participants are clustered according to when they made the decision to cross.

In order to analyze the counts in these categories, scenario 1 can be compared with scenario 3, as well as scenario 2 with 4 using the $\chi^{2}$ test, or alternatively, if in any of the cell counts are below 5, using the Fisher’s test. For both pairs the null hypothesis cannot be rejected (*p* = 0.127 and *p* = 0.054, respectively) proving that there is no significant relationship between these scenarios. In other words, the differences between the scenarios, namely speed and timing of the LED signals (either green or red) play a role.

The results show that the majority of people indicated they would cross in the time windows when it is actually safe to cross. Furthermore, in scenario 2, 80% of participants crossed when it was “expected”, i.e., when the vehicle stopped at the crossing and turned its signal from red to green. This proportion was slightly lower for scenario 4, mainly due to the time available for the decision being half of that of scenario 2.

In order to check whether the time required to make the decision to cross in the four scenarios is normally distributed, Shapiro–Wilk tests were performed. In all the four cases, the *p*-value was less than 0.05, showing that the data are not normally distributed. Therefore, to test whether there are significant differences within groups in terms of age and gender, a non-parametric test is needed. For pairwise comparisons, the Wilcoxon rank sum test can be used.

**Figure 4:**
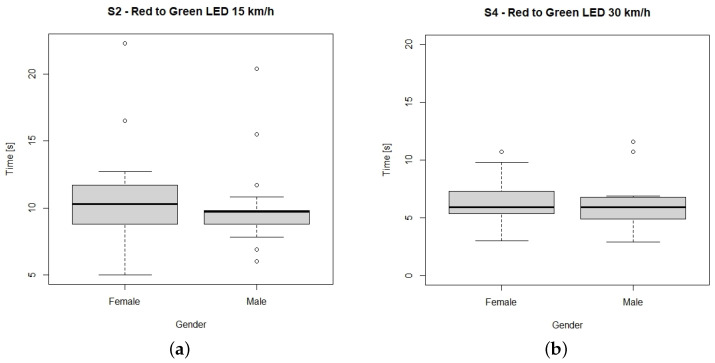
Time required to make the decision to cross by gender. (**a**) Scenario 2, (**b**) Scenario 4.

Scenarios 2 and 4 were further analyzed in terms of gender and age. Even though the male subjects made their decision to cross slightly faster (Figure 4), no significant differences were found in the decision making by gender. Subjects were also categorized in terms of their age into three clusters (<24, 16 subjects; 25–49, 20 subjects; >50, 15 subjects) and their decision making was not influenced by age (no significant difference was found).

The results of the questionnaire survey showed that participants would moderately rely on the communication with an LED light display. Overall, 75% of participants said that they would moderately trust and 18% of them would definitely trust LED communication.

## 5. Discussion and Conclusions

In our experiment, LED signals of red and green color mounted on a virtual AV, as well as two speed categories were used to investigate the decision making of pedestrians at a dedicated crossing. The majority of previous studies concluded that implicit communication is advised against text-based and instructive eHMIs [2]. Therefore, we opted for simple and explicit communication patterns. We think that our research contributed to the selection of the position and type of signal indicating the intention of the AV. As the reaction of the participants to the LED signal was unambiguously positive in scenarios 2 and 4, it can be stated that this type of eHMI is highly accepted. The results of Guo et al. [19] showed that for 65.1% of the participants, external communication would have a positive effect on their decision making. In our study it was also confirmed by the questionnaire survey that 93% of participants were positively open to the LED light display. In our study it was also concluded that male subjects made their decision to cross slightly faster; however, gender did not play a significant role in decision making. It was found that age is not a significant factor in decision making either.

Since the four scenarios had no training phase, the first phase also took on a teaching role. To a certain extent, the large variation of the reaction time in this scenario can be explained by this phenomenon.

The four scenarios in our experiment were organized one after another in the same order, starting with two scenarios with lower speed, followed by two with higher speed. Perhaps randomizing the scenarios would have provided a more reliable evaluation of the reaction of subjects. This is a potential improvement in our further research.

Further modification of the forthcoming experiments is adding an eye tracking camera to the VR set in order to collect more precise data on the nature of pedestrians’ visual cues in their decision making. Mounting the LED light display on an actual vehicle (human-driven or AV) would open the possibility to compare VR and real-world experiments. Limitations regarding the sample size will also be resolved in our future research. Nevertheless, there are examples in the literature with even fewer participants, for instance, Madigan et al. [17] used 38 subjects in their study. Mounting an LED light display on different parts of the vehicle (e.g., on the windscreen), as well as using different communication patterns, are also potential future research directions. Data collection and transmission might include recent advances in sensing techniques as well, such as reconfigurable intelligent surface [30,31,32].

Comparing the results of the consecutive scenarios S1–S4, we found that the test participants became familiar with the new communication device quite readily. Although the LED light display mounted on the virtual autonomous car was new to them, its well-known message “red or green” resulted in a quick learning process.

## Figures and Tables

**Table 1:** Subjects clustered according to when the decision to cross was made ^1^.

	Scenario 1	Scenario 2	Scenario 3	Scenario 4
Vehicle approaches ped. crossing LED red	13/51 (t < 14.3 s)	3/51 (t < 6.3 s)	6/51 (t < 4.1 s)	2/51 (t < 3.2 s)
Vehicle stops LED green	-	41/51 (6.3 s < t < 12 s)	-	30/51 (3.2 s < t < 6.1 s)
Vehicle drives over ped. crossing LED red	-	4/51 (12 s < t < 15.9 s)	-	11/51 (6.1 s < t < 7.7 s)
Vehicle leaves the ped. crossing LED red	38/51 (t > 14.3 s)	3/51 (t > 15.9 s)	45/51 (t > 4.1 s)	8/51 (7.7 s < t < 12 s)

^1^ Number of subjects deciding to cross/total number of subjects (51), in brackets—time window (t) of the particular phase in the simulation.
